# A 7.6-nW 1-kS/s 10-Bit SAR ADC for Biomedical Applications

**DOI:** 10.3390/mi13122110

**Published:** 2022-11-29

**Authors:** Yunfeng Hu, Bin Tang, Lexing Hu, Haibo Liang, Bin Li, Zhaohui Wu, Xiaojia Liu

**Affiliations:** 1 University of Electronic Science and Technology of China, Zhongshan Institute, Zhongshan 528402, China; 2 School of Microelectronics, South China University of Technology, Guangzhou 510640, China

**Keywords:** analog-to-digital converter (ADC), energy-efficient, successive approximation register (SAR)

## Abstract

This paper presents a 10-bit successive approximation register analog-to-digital converter with energy-efficient low-complexity switching scheme, automatic ON/OFF comparator and automatic ON/OFF SAR logic for biomedical applications. The energy-efficient switching scheme achieves an average digital-to-analog converter switching energy of 63.56 *CV*_ref_^2^, achieving a reduction of 95.34% compared with the conventional capacitor switching scheme for CDACs. With the switching scheme, the ADC can lower the dependency on the accuracy of *V*_cm_ and complexity of DAC control logic and DAC driver circuit. Moreover, dynamic circuits and automatic ON/OFF technology are used to reduce power consumption of comparator and SAR logic. The prototype is designed and fabricated in a 180 nm CMOS with a core size of 500 μm × 300 μm (0.15 mm^2^). It consumes 7.6 nW at 1 kS/s sampling rate and 1.8-V supply with an achieved signal-to-noise-and distortion ratio of 45.90 dB and a resulting figure of merit of 51.7 fJ/conv.-step.

## 1. Introduction

**Figure 1:**
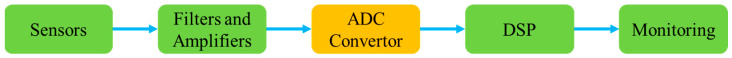
Block diagram of biomedical implantable device.

**Figure 2:**
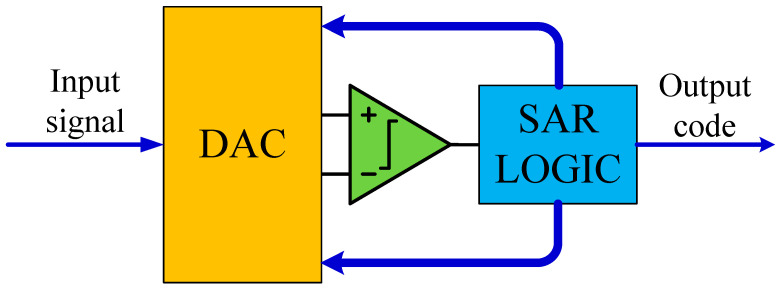
Building blocks of SAR ADC.

The development of low-power integrated circuits (ICs) will help bring portable and implantable biomedical devices and biosensors to the market. Analog front end (AFE) circuits in these products may consume most of the total power budget because they usually need to remain online to sense input signals continuously [1]. Various biomedical signals and their frequency ranges are shown in Table 1. Most biomedical signals have frequencies below 1 kHz. Figure 1 shows the basic processing units in a biomedical implantable device [2]; ADC is an intermediate unit that converts analog signals into digital signals. Successive-approximation register (SAR) analog-to-digital converter (ADC) has become an appropriate choice for low-power biomedical applications in recent years due to its low-power characteristics [2,3,4,5]. Figure 2 shows the basic components in an SAR ADC. Among the building blocks in an SAR ADC, a capacitive DAC always consumes a significant part of the total power consumption [6,7,8]. Recently, some energy-efficient switching schemes have been proposed to reduce the energy consumption of DAC capacitor arrays. [8,9,10]. Compared to conventional techniques [11], Charge-Recovery [8], Charge-Sharing [8], Capacitor-Splitting [8], Set-and-down [10], and *V*_cm_-based [9] techniques reduce the switching energy by 12.52%, 24.99%, 37.48%, 81.26%, and 87.52%, respectively. However, these schemes have various drawbacks. Capacitor-Splitting [8] and *V*_cm_-based [9] schemes have complex DAC drive circuits, the Set-and-down scheme [10] has large common-mode voltage shift, and the *V*_cm_-based scheme [9] has a high dependence on the middle reference voltage (*V*_cm_).

In this paper, the energy-efficient and low-complexity switching scheme [15] is used to realize successive approximation conversion. In the first comparison, no switching energy was consumed due to the use of top-plate sampling technology [10]. In the second comparison, no switching energy was consumed due to the closed-loop charge recycling method [16]. From the third comparison to the (*N*−1)th comparison, the reference voltage of the corresponding capacitor in the lower voltage capacitor array changes from *gnd* to *V_ref_*. In the last comparison, the reference voltage of the last capacitor in the lower voltage capacitor array changes from *gnd* to *V*_cm_. From the third comparison to the last comparison, since there is only one capacitor-switching reference voltage for each comparison, the power consumption is low. As a result, the energy-efficient and low-complexity switching scheme achieves an average switching energy of 63.56 $CV_{ref}^{2}$. Compared with the conventional switching scheme [11], this switching scheme reduces the switching energy by 95.34%. In addition, only the least significant bit (LSB) depends on the accuracy of *V*_cm_, and each capacitor only uses two reference voltages, which reduces the dependence on the accuracy of *V*_cm_ and the complexity of DAC control logic and DAC driver circuit. An automatic ON/OFF comparator is used to achieve low power consumption. The comparator consists of three parts: the automatic ON/OFF clock circuit, the dynamic preamplifier stage, and the dynamic latch stage. The automatic ON/OFF clock circuit allows the comparator to work only during comparing. Automatic ON/OFF SAR Logic consists of three parts: automatic ON/OFF clock circuit, shift control, data latch. The automatic ON/OFF clock signal is generated by the comparator output signal, and the clock is output only when the comparator is active. In order to simplify the DAC control logic and DAC driver circuit, the latch of SAR logic uses a dynamic latch with differential output. When the proposed SAR ADC uses 180 nm CMOS process and operates at a sampling rate of 1 kS/s, the ADC achieves 45.90 dB SNDR and 58.79 dB SFDR and consumes only 7.6 nW [17]. The proposed SAR ADC is suitable for portable and implantable medical sensors.

This paper is organized as follows. Section 2 describes the ADC architecture and low-power circuits. Section 3 shows the measurement results and the comparison with other ADCs. Finally, Section 4 concludes.

## 2. Proposed ADC Architecture

**Figure 3:**
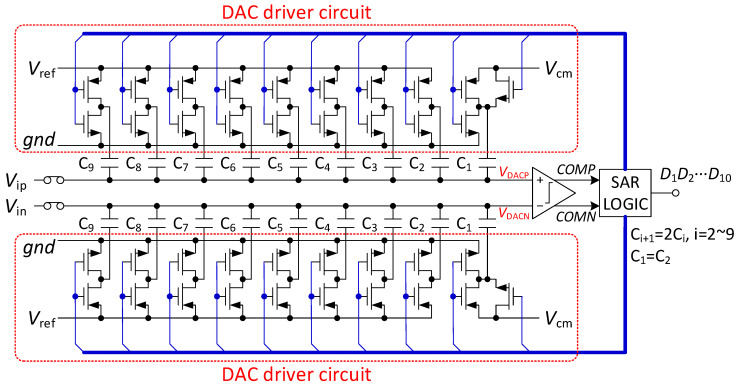
Proposed 10-bit SAR ADC architecture.

As shown in Figure 3, the proposed SAR ADC consists of comparator, SAR logic, capacitor array DAC and DAC drive circuit. Because each capacitor of the capacitor array DAC has only two reference voltages, the DAC drive circuit and DAC control logic are simple. In addition, because the last capacitor uses *V*_cm_ as the reference voltage, the number of unit capacitors is reduced by half.

### 2.1. Switching Scheme

**Figure 4:**
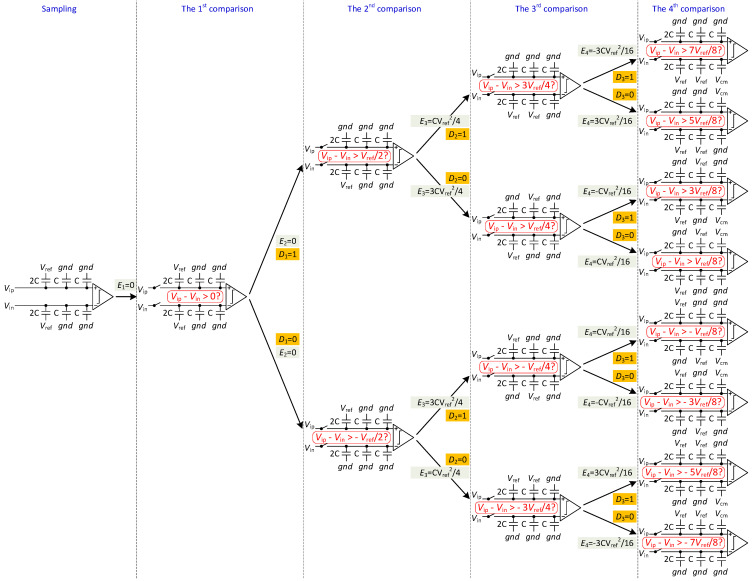
Switching procedure of 4-bit SAR DAC.

As shown in Figure 4, the operation of the switching scheme can be performed in five phases: sampling, the 1st comparison, the 2nd comparison, the 3rd to (*N*−1)th comparison, and the *N*th comparison.

Sampling: In the sampling phase, the input signals are sampled on the top-plates of all capacitors via sampling switch, with the bottom-plates of the largest capacitors connecting to *V_ref_* and other capacitors to *gnd*.

The 1st comparison: After sampling, the sampling switches are turned off. The output voltages of the DAC capacitor array are found to be

(1) $$\left\{\begin{array}{l} V_{DACP}\left(1\right)=V_{ip}\\ V_{DACN}\left(1\right)=V_{in} \end{array}\right.$$

The comparator compares the sampling signals (*V_ip_* and *V_in_*) and gets *D*_1_(MSB). No switching energy is consumed in the first comparison.

(2) $$E_{1}=0$$

The 2nd comparison (level-shift-*gnd*): If *D*_1_ = 1, the reference voltage of the largest capacitor in the positive capacitor array changes from *V_ref_* to *gnd*. If *D*_1_ = 0, the reference voltage of the largest capacitor in the negative capacitor array becomes *gnd*. As a result, the voltage of the higher side is decreased by *V_ref_*/2, and the output voltages are found to be

(3) $$\left\{\begin{array}{l} V_{DACP}\left(2\right)=V_{ip}-D_{1}\frac{V_{ref}}{2}\\ V_{DACN}\left(2\right)=V_{in}-\left(1-D_{1}\right)\frac{V_{ref}}{2} \end{array}\right.$$

The comparator compares *V_DACP_*(2) with *V_DACN_*(2) and gets *D*_2_. Due to the closed-loop charge recycling method [16], there is no switching energy consumption in the second comparison.

(4) $$E_{2}=0$$

The 3rd to (*N*−1)th comparison (“up” operation): According to the previous comparison results, the reference voltage of the corresponding capacitor in the lower voltage capacitor array is switched from *gnd* to *V_ref_*, while the other one (in the higher voltage capacitor array) remains unchanged. For example, in the third comparison, if *D*_2_ = 1, the reference voltage of the second largest capacitor in the negative capacitor array is switched from *gnd* to *V_ref_*. If *D*_2_ = 0, the reference voltage of the second largest capacitor in the positive capacitor array is switched from *gnd* to *V_ref_*. The ADC repeats the procedure until the (*N*−1)th comparison is completed. The output voltages of each comparison are found to be

(5) $$\left\{\begin{array}{l} V_{DACP}\left(i\right)=V_{ip}-D_{1}\frac{V_{ref}}{2}+\sum_{j=2}^{i-1}\left(1-D_{j}\right)\frac{V_{ref}}{2^{j}}\\ V_{DACN}\left(i\right)=V_{in}-\left(1-D_{1}\right)\frac{V_{ref}}{2}+\sum_{j=2}^{i-1}D_{j}\frac{V_{ref}}{2^{j}} \end{array}\right.$$

The comparator compares *V_DACP_*(*i*) with *V_DACN_*(*i*) and gets *D_i_*. During the switching procedure, there is only one capacitor switch for each comparison, resulting in less switching activity and lower energy. Based on the switching energy calculation method in [6], the switching energy of each comparison is found to be

(6) $$E_{i}=\left\{\begin{array}{l} 2^{N-i-1}-2^{N-2i}-D_{i-1}\sum_{j=1}^{i-2}D\left[j\right]2^{N-j-i-1}\\ -\left(1-D_{i-1}\right)\sum_{j=1}^{i-2}\left(1-D\left[j\right]\right)2^{N-j-i-1} \end{array}\right\}CV_{ref}^{2}$$

*N*th comparison: In the *N*th comparison, the reference voltage of the last capacitor in the lower side is switched from *gnd* to *V*_cm_ while the other one (on the higher side) remains unchanged. The output voltages and switching energy are found to be

(7) $$\left\{\begin{array}{l} V_{DACP}\left(N\right)=V_{ip}-D_{1}\frac{V_{ref}}{2}+\sum_{j=2}^{N-1}\left(1-D_{j}\right)\frac{V_{ref}}{2^{j}}\\ V_{DACN}\left(N\right)=V_{in}-\left(1-D_{1}\right)\frac{V_{ref}}{2}+\sum_{j=2}^{N-1}D_{j}\frac{V_{ref}}{2^{j}} \end{array}\right.$$

(8) $$E_{N}=\left\{\begin{array}{l} D_{1}\left(1-D_{N-1}\right)\left[2^{-2}-2^{-N}-\sum_{j=1}^{N-2}\left(1-D\left[j\right]\right)2^{-j-1}\right]\\ +\left(1-D_{1}\right)D_{N-1}\left[2^{-2}-2^{-N}-\sum_{j=1}^{N-2}D\left[j\right]2^{-j-1}\right] \end{array}\right\}CV_{ref}^{2}$$

The average switching energy of the switching scheme is derived as

(9) $$E_{average}=\bar{\sum_{D_{1}D_{2}\cdots D_{N}=00\cdots 0}^{11\cdots 1}\left(\sum_{i=1}^{N}E_{i}\right)}=\left(2^{N-4}-2^{-1}+2^{-4}\right)CV_{ref}^{2}$$

**Figure 5:**
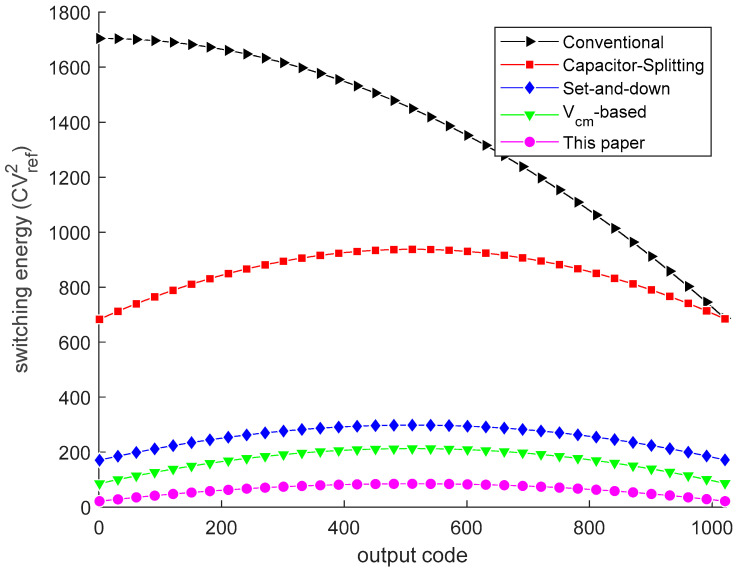
Switching energy against output codes. The black [11], red [8], blue [10], green [9], and magenta curves are switching energy.

**Figure 6:**
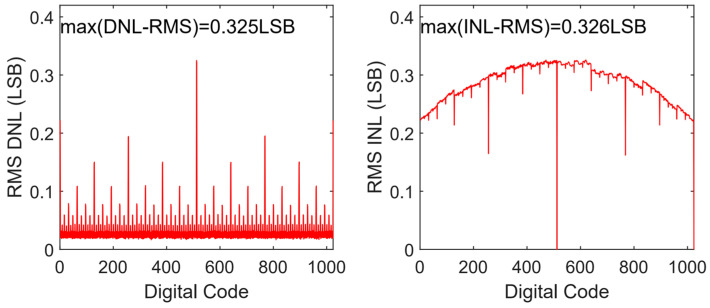
DNL and INL versus output code of the proposed switching scheme.

Figure 5 shows switching energy at each output code for different switching schemes. The average switching energy of the switching scheme used for 10-bit SAR ADC is 63.56 $CV_{ref}^{2}$. Compared with the conventional switching scheme [11], the used switching scheme [15] and Capacitor-Splitting [8], Set-and-down [10], and *V*_cm_-based [9] schemes reduce the switching energy by 95.34%, 37.48%, 81.26%, and 87.52%, respectively. Figure 6 presents the 500-run Monte Carlo simulation results of the proposed DAC switching scheme with unit capacitor mismatch of *σ*_u_/*C*_u_ = 1%. The RMS DNL and the RMS INL of the proposed DAC switching scheme are 0.325 LSB and 0.326 LSB, respectively.

### 2.2. Automatic ON/OFF Comparator

**Figure 7:**
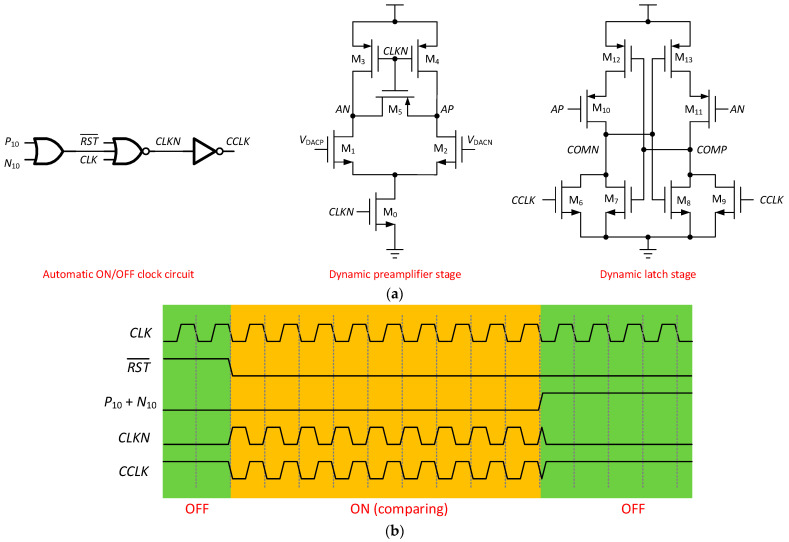
Automatic ON/OFF comparator. (**a**) Schematic diagram; (**b**) Timing diagram.

A low-power two-stage full dynamic comparator is reported in [18]. In order to save more power consumption of the comparator, an automatic ON/OFF clock circuit is added to the comparator. As shown in Figure 7a, the comparator consists of automatic ON/OFF clock circuit, dynamic preamplifier stage, and dynamic latch stage. In the dynamic preamplifier stage, *V*_DACP_ and *V*_DACN_ are the output signals of the DAC capacitor array and are connected to the differential inputs of the comparator. *AP* and *AN* are differential outputs of the dynamic preamplifier stage. In the dynamic latch stage, *COMP* and *COMN* are the comparison results, which are obtained by *AP*, *AN*, and *CCLK* driving the latch. In the process of result latching, no power-to-ground current path is formed, so the comparator only has a dynamic power supply. The automatic ON/OFF clock circuit generates the clock for comparator operation. When the $\bar{RST}$ is high or P_10_ + N_10_ (P_10_ and N_10_ are the 10th comparison result that latched in SAR logic) is high, there is no clock output, the comparator is in the OFF state, and the comparator has no power consumption. Figure 7b shows the timing diagram of the comparator.

### 2.3. Automatic ON/OFF SAR Logic

**Figure 8:**
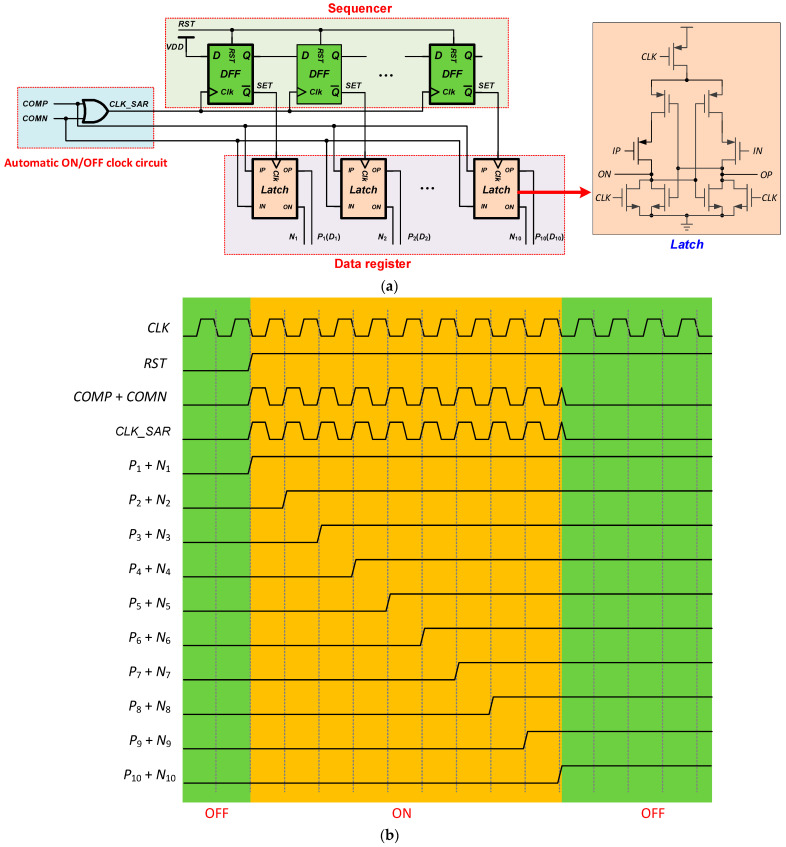
Automatic ON/OFF SAR logic. (**a**) Block diagram; (**b**) Timing diagram.

As shown in Figure 8, the automatic ON/OFF SAR logic consists of an automatic ON/OFF clock circuit, a sequencer, and a data register. The sequencer is a shift register that shifts the set signal through a series of D flip-flops. The set signal is then used to activate the Latch in the data register. When the last D flip-flop in the sequencer is triggered, the sequencer will be reset and await the next conversion cycle. The data register is composed of dynamic latches, which can latch the differential outputs of the comparator and output differential data. Differential output makes DAC logic circuit simpler. The automatic ON/OFF clock circuit is used to provide the drive clock signal for the shift register. Drive clock is only ON while comparison results are being latched, thus reducing SAR logic power consumption. Figure 8b shows the timing diagram of the SAR logic.

### 2.4. DAC Driver Circuit

**Figure 9:**
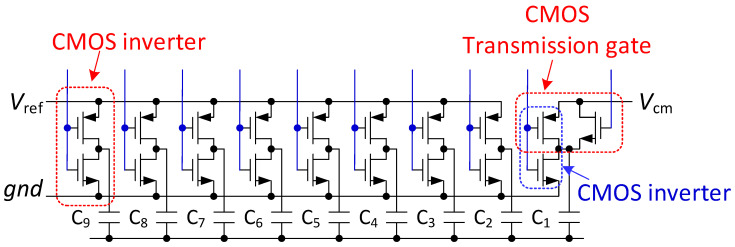
DAC driver circuit.

As shown in Figure 9, each capacitor requires two reference voltages. The reference voltages of C_2_ to C_9_ capacitors are *V_ref_* and *gnd*, and the drive circuit can be realized by CMOS inverter. The reference voltage of C_1_ capacitor is *V*_cm_ and *gnd*, and the driving circuit adopts a hybrid structure of CMOS transmission gate and CMOS inverter circuit.

### 2.5. Capacitor Array

**Figure 10:**
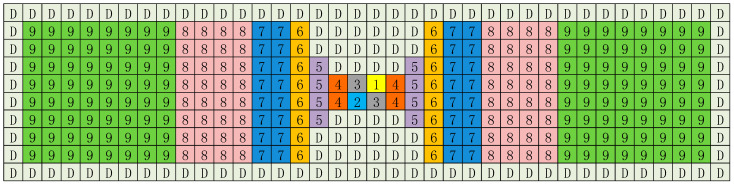
The layout floorplan of the DAC capacitor array.

Figure 10 illustrates the floorplan of the capacitor array DAC for a single side. Both sides have identical layout design. DAC capacitors and dummy capacitors are represented by squares (unit capacitors) in different colors. DAC capacitors are surrounded by dummy capacitors to minimize the proximity effects and second-order lithographic errors. Additionally, a common centroid layout is used to reduce parasitic effects.

## 3. Results

**Figure 11:**
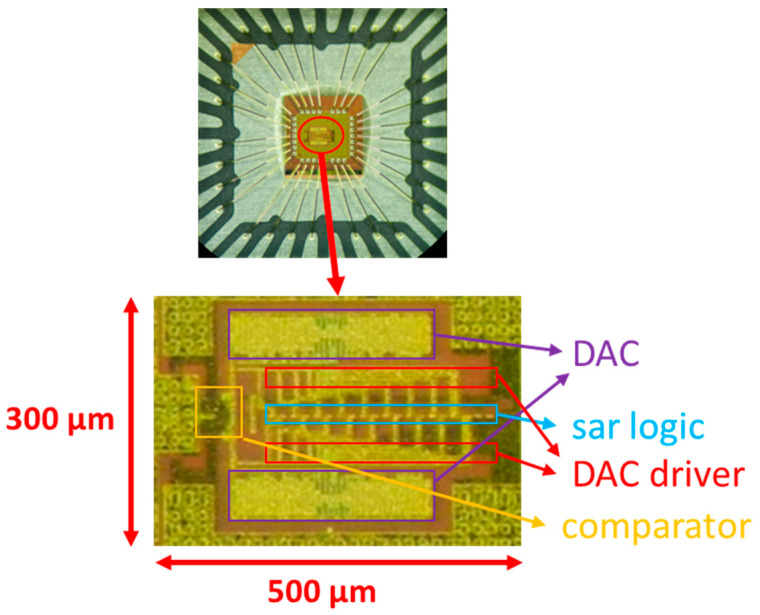
Chip micrograph.

**Figure 12:**
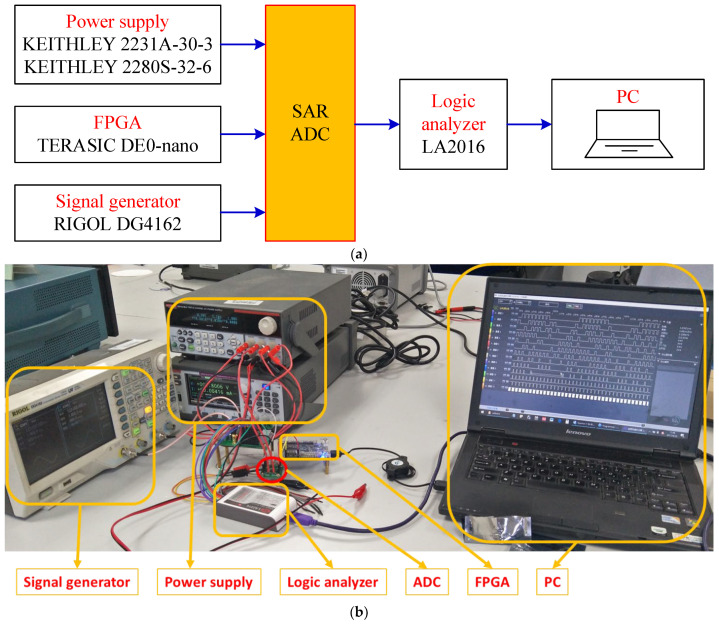
Measurement environment. (**a**) Block diagram; (**b**) Photo.

**Figure 13:**
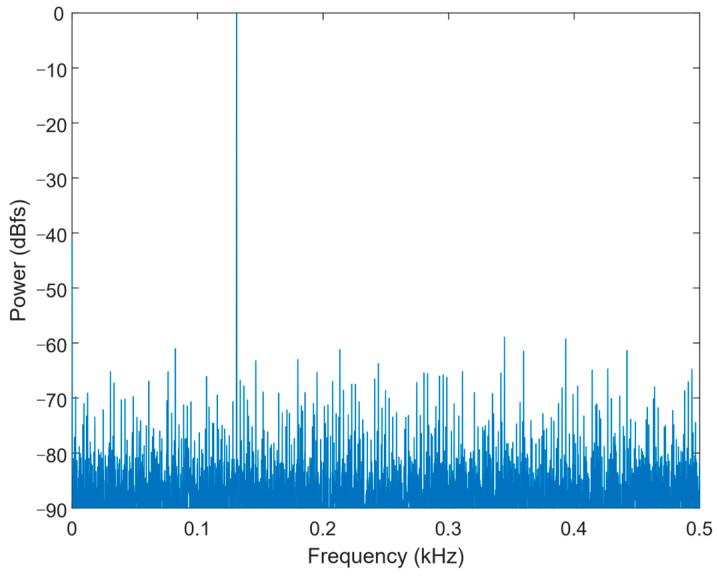
FFT spectrum.

Figure 11 shows the chip micrograph of the ADC prototype fabricated in 180 nm CMOS process with a core area of 0.15 mm^2^ (500 μm × 300 μm). Figure 12 shows the measurement environment. The power supply device provides power and reference voltage for the SAR ADC chip. The FPGA generates timing signals to control the work of SAR ADC chip. The signal generator generates a differential sinusoidal signal and connects to the analog signal input of the SAR ADC chip. The logic analyzer is connected to the digital signal output of the SAR ADC chip and collects the output data. The PC takes out the measured data from the logic analyzer and then analyzes various performance indicators of the chip. As shown in Figure 13, the signal-to-noise-and-distortion ratio (SNDR) is 45.15 dB, and the effective number of bits (ENOB) is 7.2 bit at 1.8 V supply and 1 kS/s sampling rate. The SAR ADC consumes 7.6 nW, and the calculated FoM is 51.7 fJ/conv.-step. The Figure-of-Merit (*FoM*) was calculated from the following equation:

(10) $$FoM=\frac{Power}{2^{ENOB}\times f_{samping}}$$

Table 2 shows the performance comparison between the proposed ADC and other ADCs. Compared with another 180 nm ADC, the proposed ADC has a convincing performance. If the proposed SAR ADC uses the 65 nm process, it may have better performance.

## 4. Conclusions

This paper has presented a low-power SAR ADC for biomedical applications. The ADC uses an energy-efficient, low-complexity switching scheme to reduce power consumption. Because of the top-plate sampling and level-shift-gnd operations, the switching scheme did not consume energy in the first and second comparisons. Thanks to the use of *V*_cm_ for the last capacitor, the total capacitance is reduced by half, so the energy consumption of DAC is also reduced. In addition, because of the automatic ON/OFF technology, the comparator and SAR logic only generate energy consumption during operation. The proposed SAR ADC achieves FoM of 51.7 fJ/conv.-step at 1.8 V supply and 1 KS/s sampling rate. If the ADC adopts a low-voltage design method, more energy consumption will be saved. The proposed low-power SAR ADC is suitable for biomedical applications.

## Figures and Tables

**Table 1:** Frequency ranges of various biomedical signals.

Biomedical Signals	Frequency Range
ECG [2]	0.05–100 Hz
ECoG [12]	70–110 Hz
EMG [13]	50–150 Hz
EEG [14]	0–100 Hz

**Table 2:** Performance comparison.

Specification	[19]	[20]	[21]	This Work
Technology (nm)	65	180	65	180
Resolution (bit)	14	10	8	10
Supply Voltage (V)	0.8	1	0.6	1.8
Sampling Rate (kS/s)	10	1	0.5	1
Power (nW)	1980	120	1.8	7.6
ENOB (bit)	12.5	9.76	7.14	7.2
FoM (fJ/conv.-step)	34.2	138.4	25.5	51.7

## Data Availability

Not applicable.
